# Evaluation of barometric whole-body plethysmography for therapy monitoring in cats with feline lower airway disease

**DOI:** 10.1371/journal.pone.0276927

**Published:** 2022-10-27

**Authors:** Hannah Gareis, Lina Hörner-Schmid, Yury Zablotski, Jelena Palić, Bianka Schulz

**Affiliations:** 1 Clinic of Small Animal Medicine, Ludwig Maximilian University of Munich, Munich, Germany; 2 Division of IDEXX Laboratories, Vet Med Labor GmbH, Kornwestheim, Germany; University of Western Ontario, CANADA

## Abstract

**Objectives:**

Feline lower airway disease (FLAD) is a common respiratory condition in cats. Traditionally, response to therapy is monitored only by evaluation of clinical signs and radiographic examination of the lungs. Barometric whole-body plethysmography (BWBP) is considered a non-invasive, well-tolerated form of measuring airway reactivity in cats. The aim of the study was to assess pulmonary function testing by BWBP for non-invasive evaluation of response to therapy in cats with FLAD and to investigate whether BWBP parameters correlate with clinical severity.

**Material and methods:**

The prospective study included 25 client-owned cats, diagnosed with FLAD on the basis of their medical history, clinical signs, radiographic findings, and bronchoalveolar lavage fluid (BALF) examination. At three time points (day 0, 14, and 60), a standardised owner questionnaire, a clinical examination and BWBP measurements were carried out. Results of the questionnaire and the clinical examination were evaluated using a clinical 12-point score. Individual therapy was administered to all patients after diagnosis, based on the severity of disease and compliance of the cat.

**Results:**

The total clinical score significantly improved over the entire study period (p<0.001). Significant improvement was detected for the frequency of coughing (p = 0.009), respiratory distress (p = 0.001), lung auscultation findings (p = 0.002), and general condition and appetite (p = 0.045). The BWBP parameter *Penh*, an indicator of bronchoconstriction, improved significantly under initial therapy between day 0 and 14 (p = 0.009). A significant correlation between *Penh* and the severity of auscultation findings was seen on day 0 (r = 0.40; p = 0.013).

**Conclusion:**

The study supports the role of *Penh* as a non-invasive parameter for monitoring initial treatment response in cats with FLAD. Further studies are needed to address whether other BWBP parameters might be suitable for non-invasive therapy monitoring of FLAD. Clinical evaluation is always essential in cats with FLAD to evaluate treatment response.

## Introduction

Feline lower airway disease (FLAD) is considered the most common lower respiratory condition in cats [1] and occurs in approximately 1–5% of the adult cat population [2]. Depending on the predominant type of inflammatory cells in the airways, a distinction is made between feline asthma (FA) and chronic bronchitis (CB). FA is primarily characterised by eosinophilic and CB by neutrophilic airway inflammation [3, 4]. Recent studies suggest a third category of FLAD, characterised by mixed eosinophilic and neutrophilic inflammation [5–8]. Nevertheless, the diagnostic features, treatment options and prognosis of different FLAD conditions commonly overlap [2].

The exact pathology underlying FLAD is not yet fully understood [9]. The aetiology of FA is believed to be primarily of allergic origin and based on a type-1-hypersensitivity response to inhaled allergens within the airways. This aeroallergen-induced stimulation leads to a response of type-2 CD4-positive T-helper cells. CD4-positive lymphocytes secrete cytokines, including interleukin-5, which in turn promotes the formation and activation of eosinophils in the respiratory tract and causes pathological changes [2, 3, 10–14]. As a result, airway hyperresponsiveness, bronchoconstriction and permanent architectural remodelling (“airway remodelling”) occur [14].

In addition to inflammation of the bronchial mucosa and excessive mucus secretion, hypertrophy and constriction of the smooth muscles in the airways are key features of FLAD, leading to airway obstruction. This results in typical clinical signs, such as coughing, wheezing and episodes of respiratory distress, and occasional sneezing, in varying degrees of severity [2, 11, 14–19]. These alterations of the airways can lead to deterioration of pulmonary function [20].

Establishing a diagnosis of FLAD requires assessment of different diagnostic features, including medical history, clinical examination, haematology, serum biochemistry, thoracic radiographs or computed tomography, exclusion of parasitic respiratory disease, evaluation of bronchoalveolar lavage fluid (BALF) including cytology and microbiology, and response to appropriate therapy [1, 10, 21]. Treatment of FLAD aims at controlling clinical signs and reducing airway inflammation. Corticosteroids alone or in combination with bronchodilators are most commonly used for this purpose [2, 9, 12–14, 20]. In this context, corticosteroids represent the most effective long-term treatment of FLAD. An important effect of corticosteroids is the inhibition of the expression of genes responsible for cytokine production, which induce airway inflammation [2]. Due to adverse side effects [22], which can accompany long-term administration of high-dose oral steroids in cats, inhaled administration of corticosteroids is an alternative option for long-term treatment [23–26]. Traditionally, therapeutic response in cats with FLAD is monitored by evaluation of clinical and radiographic parameters only [3, 9, 16, 20]. It is well known, however, that airway inflammation in humans with asthma can persist even in the absence of clinical signs [27–30]. Subclinical respiratory inflammation has also been demonstrated in cats [20]. Additionally, cats with FLAD do not always present abnormal radiographic findings [16]. Therefore, clinical and radiographic assessment of therapeutic response also involves a certain degree of subjectivity [31]. In humans with inflammatory bronchial disease, pulmonary function testing by spirometry [32–34] and measurement of nitric oxide in exhaled air [35, 36] are commonly used for objective evaluation and monitoring of therapeutic response. As active cooperation is naturally limited in cats, pulmonary function tests used in human medicine cannot be directly transferred to use in veterinary patients [9, 34]. An alternative non-invasive method for pulmonary function testing in awake, spontaneously breathing cats is barometric whole-body plethysmography (BWBP). BWBP is a well-tolerated procedure for assessing respiratory reactivity in cats with naturally occurring FLAD [4, 37–41] and it is easily repeatable due to its non-invasive nature [42, 43].

Historically, barometric plethysmography was developed for use in human infants [44] and was modified for veterinary patients at a later stage [45]. In BWBP, the awake and non-restrained cat is placed in an airtight plexiglass chamber, which is ventilated with a bias flow [43, 45, 46]. While the cat is breathing spontaneously, the pressure changes generated by inspiration and expiration are recorded [45, 47]. These pressure changes produce respiratory waveforms [42], referred to as pseudo-flow and pseudo-volume estimates, because, in contrast to spirometry, they do not directly measure airflow at the level of nose and mouth [43, 45, 48]. By adding a bronchoprovocator as part of a respiratory reactivity test, the sensitivity of BWBP can be increased [43]. A previous study demonstrated that BWBP can be used to discriminate between healthy cats and cats with FLAD [39]. Other studies indicated that BWBP can potentially be used to discriminate between neutrophilic and eosinophilic airway inflammation in cats [4, 41]. Allerton and coworkers [4] reported a correlation between the level of granulocytes in the lower airways of cats with FLAD and the BWBP parameters *Penh* (indicator of bronchoconstriction) and *C-Penh-300* (carbachol concentration inducing a 300% increase of post-saline *Penh* during bronchoprovocation). The ratio *PEF/EF25* in cats with naturally occurring FLAD, calculated from measured BWBP parameters, also correlated with the level of granulocytes in BALF [38].

The aim of the study was to find out whether pulmonary function testing by BWBP can be used for non-invasive evaluation of therapeutic response in cats with naturally occurring FLAD and to investigate whether certain BWBP parameters correlate with clinical severity.

## Material and methods

### Ethical note

The prospective observational study was approved by the Ethics Committee of the Centre for Clinical Veterinary Medicine of Ludwig Maximilian University (LMU) of Munich (139-20-07-2018). Written informed consent was obtained from the owner or legal custodian of all animals for the procedures undertaken.

### Study population

Client-owned cats with clinical signs suggestive of FLAD, such as chronic cough, episodes of wheezing and/or respiratory distress, which were presented to the LMU Clinic of Small Animal Medicine for diagnostic workup between May 2018 and July 2021, were eligible for inclusion in the study. Cats were not included if they were not stable enough for a diagnostic work-up or if cardiac or pleural space disease or respiratory disease other than FLAD was detected, based on clinical, radiographic or BALF-examination findings. In addition, cats that had received antibiotic therapy within 14 days prior to presentation were excluded. A total of 25 cats, diagnosed with FLAD on the basis of medical history, clinical, radiographic and BALF examination, were included in the study.

### Study design

All cats were presented at three examination time points (day 0, 14 and 60). At the first presentation (day 0), a standardised owner questionnaire, modified from an earlier study [49], was used to obtain medical history. In addition, a detailed clinical examination was performed. Based on the findings of the medical history and the clinical examination, each cat was assigned a clinical score based upon a previously published 12-point score established for cats with FLAD [39]. After clinical examination, BWBP was carried out before further examinations were performed. In addition, haematology, serum chemistry and thoracic radiographs in two dimensions (laterolateral and ventrodorsal or dorsoventral) were obtained. Examination of a faecal sample using the Baerman-Wetzel technique was performed in six cats to exclude lungworm infection, as they were cats with access to the outdoors. As no cat originated from an endemic area for heartworm infection, and heartworm infection is not endemic in Germany, testing for *Dirofilaria immitis* was not performed. Bronchoalveolar lavage (BAL) was carried out in all cats under anaesthesia as previously described [50]. BAL was obtained blindly (n = 24) or under endoscopic visual control (n = 1). Prior to BAL, all cats received a one-time injection of Terbutaline 0.01 mg/kg SC to decrease the risk of bronchoconstriction during the procedure. Patients were anaesthetised using various protocols according to ASA classification, and intubated with a sterile endotracheal tube. BAL was performed with 3–4 ml of sterile, isotonic saline solution (0.9%, B. Braun Vet Care) applied into a sterile polyvinyl chloride catheter (CH 4.5, 1.0 × 1.5; B. Braun Vet Care). A mechanical vacuum device was used to retrieve the solution. If necessary, a second BAL was performed using the same principle. Collected samples were centrifuged and smears were stained with modified Wright’s stain for cytological evaluation. The cell count of BALF was determined in each sample before centrifugation. Cytological examination of all smears was carried out by the same certified clinical pathologist (JP). For this purpose, at least two direct smears and one to two cytospin preparations were examined in each case. Per slide, 100 inflammatory cells were differentially counted in several microscopic fields and classified based on the cell types present: eosinophilic inflammation (≥17% eosinophils, <7% neutrophils), neutrophilic inflammation (≥7% neutrophils, <17% eosinophils) and mixed inflammation (≥7% neutrophils and ≥17% eosinophils) [8].

In addition, each BALF was submitted for aerobic bacterial culture to the LMU Institute of Infectious Diseases and Zoonoses, Munich, Germany, and PCR for *Mycoplasma* species was performed on BALF of 24 cats (Synlab Group or Idexx Laboratories).

Each cat was presented for follow-up examinations on day 14 and 60. At both time points, the clinical 12-point score was obtained on the basis of the information from the owner questionnaire and clinical examination, and BWBP was reperformed.

### Clinical 12-point score

Each cat was assigned a total clinical score in accordance with the published clinical 12-point score at all three examination time points. The score was established based on the information derived from the standardised owner questionnaire and the findings of the clinical examination. The total clinical score comprised of coughing frequency, frequency of respiratory distress, findings of lung auscultation, as well as general condition and appetite of the cat (Table 1): Thus, the lower the cat’s total clinical score, the better the clinical condition. By determining the total clinical score at all three examination time points, the clinical progression under therapy was assessed.

**Table 1:** Clinical 12-point score in cats with FLAD.

Clinical parameter	Clinical 12-point score
**Coughing frequency**	0 = absent
	1 = occasional cough (less than once per month)
	2 = infrequent cough (at least once per month)
	3 = frequent cough (at least once per week, at least one day)
	4 = intensive cough (at least every 1–2 days)
	5 = very intensive cough (several times per day)
**Frequency of respiratory distress**	0 = no respiratory distress
	1 = respiratory distress only after excitement or stress
	2 = moderate respiratory distress (breathing difficulty observed more than once at home at rest)
	3 = severe respiratory distress (permanently present at rest)
**Thoracic auscultation**	0 = no abnormal sounds
	1 = increased respiratory sounds
	2 = abnormal sounds such as crackling or wheezing
**General condition and appetite**	0 = normal general condition and appetite
	1 = lethargic/hyporexic
	2 = unresponsive/anorexic
**Total clinical score**	= Addition of all four scores

The total clinical score is calculated from the individual criteria of the clinical 12-point score. The lower the total clinical score, the better the clinical condition.

### Barometric whole-body plethysmography

The pulmonary function of all cats was measured using a whole-body plethysmograph (Buxco® FinePoint Small Animal Whole Body Plethysmograph, Data Science International (DSI), New Brighton, Minnesota, USA) at all three study time points. A transparent plexiglass chamber was ventilated with a continuous air flow using bias flow (Buxco® Multi-function Bias Flow, Data Science International (DSI), New Brighton, Minnesota, USA). The chamber was equipped with sieve pneumotachographs with a known air resistance which allowed air flow in and out of the chamber. An attached pressure transducer (Halcyon™ pneumotach, Data Science International (DSI), New Brighton, Minnesota, USA) measured the pressure changes in the chamber and allowed the flow rate “box flow” to be derived. The pressure transducer was connected to a data acquisition card with a preamplifier (Buxco® QT Digital Preamplifier, Data Science International (DSI), New Brighton, Minnesota, USA). The chamber signal was passed on to a computer with an associated software program (Buxco® FinePoint Small Animal Whole Body Plethysmograph, Data Science International (DSI), New Brighton, Minnesota, USA) to document the pressure changes in real time. By applying Boyle’s law to the “box flow”, the BWBP parameters were measured.

Before starting each measurement, the system pressure was calibrated by applying 50 ml of room air into the chamber. Each cat was placed in the plexiglass chamber awake and in a conscious state. After 20 minutes of acclimatisation time, the actual measurement of 10 minutes’ duration was recorded.

The following parameters were determined during the BWBP measuring period: respiratory rate (*RR*; breaths/min), inspiratory time (*Ti*; s), expiratory time (*Te*; s), tidal volume (*TV*; ml), minute volume (*MV*; ml/min), peak inspiratory pseudo-flow (*PIF*; ml/s), peak expiratory pseudo-flow (*PEF*; ml/s), expiratory flow at end-tidal volume plus 50% tidal volume (*EF50*; ml/s), relaxation time (*RT*; ms; time at which 65% of tidal volume is exhaled), inspiratory end-pause (*EIP*; ms), expiratory end-pause (*EEP*; ms) and indices of bronchoconstrictor pause (*PAU*; unitless; (*Te*-*RT*)/*RT*) and enhanced pause (*Penh*; unitless; (*PEF*/*PIF*) x (*Te*-*RT*)/*RT*). Artefactual waveforms caused by sniffing, vocalisation and body movements were automatically erased by the software. As *TV*, *MV*, *PIF*, and *PEF* are affected by the cat’s body weight, these parameters were subsequently divided by body weight: tidal volume per body weight (*TV/BW*; ml/kg), minute volume per body weight (*MV/BW*; ml/min/kg), as well as peak inspiratory and peak expiratory pseudo-flow per body weight (*PIF/BW* and *PEF/BW*; ml/s/kg). The quotient of peak expiratory pseudo-flow and expiratory flow at end expiratory volume plus 50% tidal volume (*PEF/EF50*; unitless) was calculated.

### Treatment

Each cat was treated individually according to severity of the disease and compliance of the cat and the owner. Three cats received a single injection of dexamethasone (0.4 mg/kg IV) after BAL due to difficult recovery.

The selected therapy for FLAD in this study is shown in Table 2. A list of all therapeutic agents for each cat is given in S1 Table. The starting time point of the therapy varied, depending on whether the cat required therapy straight after BAL sampling due to the severity of the disease, or whether the findings of the BALF examinations and thus a diagnosis could be awaited. 2/25 cats did not receive therapy until day 14, because the owners did not want to revisit the clinic between examination time points to start therapy, as these patients were in a clinically stable condition.

**Table 2:** Individual therapy in cats with FLAD between study time points.

Therapeutic agents	Day 0 –Day 14	Day 14 –Day 60
**Prednisolone only**	7/25	1/25
**One-time Dexamethasone + Prednisolone**	2/25	0/25
**One-time Dexamethasone + Prednisolone + Terbutaline**	1/25	0/25
**Prednisolone + Terbutaline**	4/25	0/25
**Prednisolone + Fluticasone propionate 250 μg**	2/25	4/25
**Prednisolone + Terbutaline + Fluticasone propionate 250 μg**	3/25	0/25
**Prednisolone + Budesonide 200 μg**	1/25	1/25
**Cyclosporine + Fluticasone propionate 125 μg**	1/25	1/25
**Terbutaline + Fluticasone propionate 250 μg**	1/25	0/25
**Salmeterol and fluticasone propionate 25 μg/125 μg only**	0/25	1/25
**Fluticasone propionate 250 μg only**	0/25	17/25
**Terbutaline only**	1/25	0/25
**No therapy**	2/25	0/25

Initial treatment consisted of the administration of systemic anti-inflammatory therapy in 22/25 cats; in 21/22 cats this initial therapy was started between day 0 and day 14. It involved Prednisolone 1 mg/kg (IQR 1–1 mg/kg) every 24 hours (q24) PO in 21/25 cats and Cyclosporine (Sporimune® 5 mg/kg q24 PO) in 1/25 cats. Systemic anti-inflammatory therapy was combined directly with inhaled glucocorticoids in 7/22 cats and with a bronchodilator, Terbutaline 0.06 mg/kg +/- 0.03 mg/kg (range: 0.01–0.1 mg/kg) q8-q12 PO, in 8/22 cats. 3/22 cats initially received a combination of Terbutaline and systemic and inhalative glucocorticoids.

3/25 cats were treated only with the inhalative form of anti-inflammatory therapy over the entire study period. 2/3 cats received Terbutaline initially, prior or parallel to the start of inhalation therapy.

The duration of the initial therapy was based on the severity of the disease, the clinical response to therapy, and the duration of the time period the cat needed to adapt to inhalation therapy. Based on these factors, Terbutaline and the systemic anti-inflammatory agent were phased out after initial therapy, if possible, and at the same time a conversion to inhaled glucocorticoids was aimed for. As inhaled glucocorticoids were intended to be the sole therapeutic agent for long-term therapy in all cats, 24/25 cats received inhalation therapy until the end of the study period. Between day 14 and day 60, it was possible for 18/25 cats to receive inhalation therapy only. 7/25 cats required further systemic anti-inflammatory therapy during this period, whereby the Prednisolone therapy was in the process of being tapered off in 5/7 cats.

Inhalative agents administered in this study were fluticasone propionate (Flutide® 250 μg; one puff q12) in 21/24 cats, fluticasone propionate (Flutide® 125 μg; one puff q12) in 1/24 cats, salmeterol and fluticasone propionate (Serroflo® 25 μg/125 μg; one puff q12) in 1/24 cats or budesonide (Budiair® 200 μg; one puff q12) in 1/24 cats. Inhalation therapy was performed using a spacing chamber with oronasal mask (Aerokat®; Trudell Medical International).

### Statistical analysis

The statistical evaluation was performed with SPSS version 28.0.1.0 software. BWBP measurements were extracted into Microsoft Office Excel files and the values of 10-minute measuring periods were subsequently averaged. The Shapiro-Wilk test was applied to test for normal distribution, and the data were presented as mean +/- standard deviation for normally distributed data or median with interquartile range (IQR) for not-normally distributed data. The total clinical score, the individual criteria of the clinical 12-point score, and the individual BWBP parameters were compared on the basis of not-normally distributed data by Friedman-rank-sum test at all three time points. Subsequently, all p-values of post-hoc tests (i.e. Conover’s tests) were corrected with the Bonferroni method for multiple comparisons.

Each BWBP parameter was evaluated for its correlation to the clinical 12-point score using Kendall’s rank correlation coefficient.

The level of significance was set at α <0.05. Then, the Bonferroni-adjusted significance level for multiple comparisons was calculated to be α <0.017 as there were three tests (0.05/3 = 0.017).

## Results

### Study population

**Fig 1:**
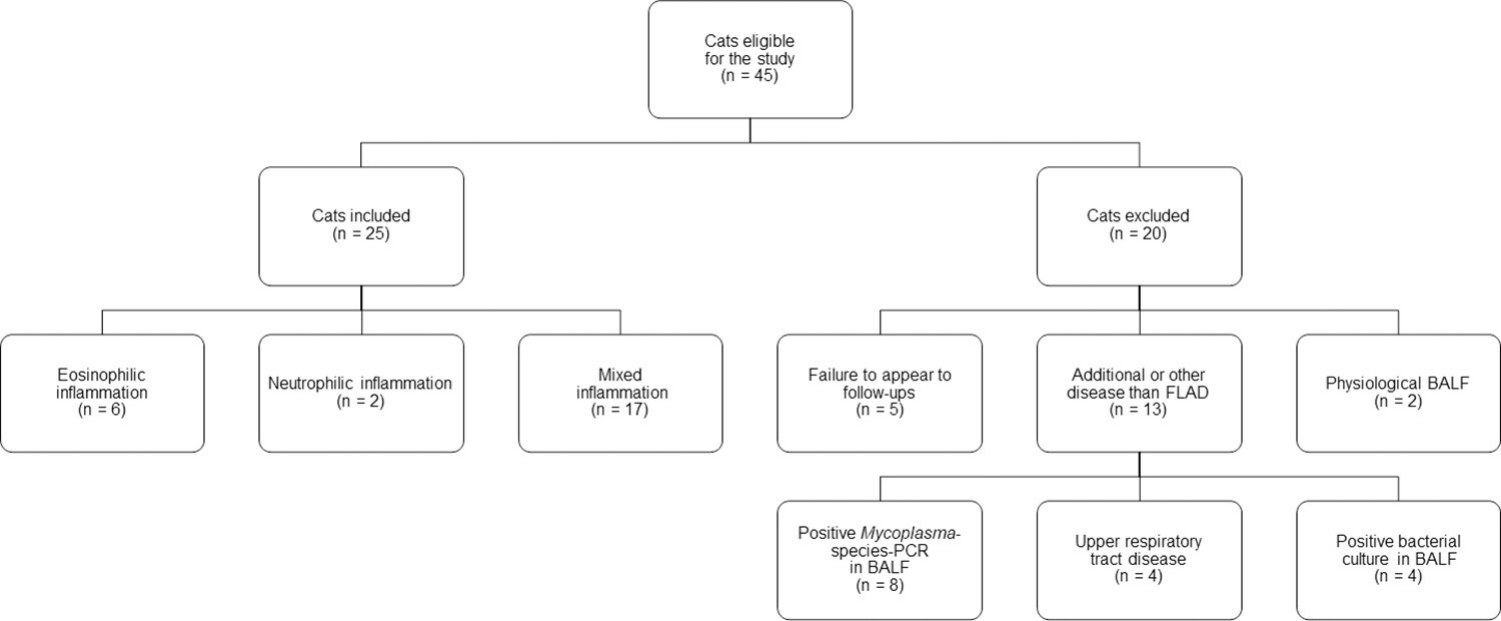
Flow chart for selection and grouping of study participants.

Forty-five cats were eligible for participation in the study (Fig 1). Twenty cats were excluded from the study for one or more of the following reasons: presence of upper respiratory tract disease (n = 4), positive *Mycoplasma*-species-PCR in BALF (n = 8), positive bacteriological culture of BALF (n = 4), physiological BALF cytology (n = 2) or failure to appear for follow-up appointments (n = 5).

A total of 25 cats was included in the study. Breeds included European Shorthair (n = 9), European Shorthair mix (n = 2), Abyssinian (n = 2), Ragdoll (n = 2), Siamese mix (n = 2), Siamese (n = 1), British Shorthair (n = 1), Bengal (n = 1), Siberian Forest (n = 1), Turkish Van (n = 1), Maine Coon (n = 1), Maine Coon mix (n = 1) and Norwegian Forest mix (n = 1). There were 13 neutered females, nine neutered males, two intact males and one intact female cat. The mean age at first presentation was 5 years +/- 3 years (range: 1 year–13 years), and the mean body weight was 4.8 kg +/- 1.4 kg (range: 2.8 kg–8.3 kg). The study population consisted of 12 indoor cats, 10 cats with access to a balcony or patio, two outdoor cats and one shelter cat.

### Bronchoalveolar lavage cytology

Based on the cytological examinations, six cats were diagnosed with eosinophilic inflammation (EI), two cats with neutrophilic inflammation (NI) and 17 cats with mixed inflammation (MI). The cytological findings of BALF of all cats are shown in Table 3.

**Table 3:** Cytologic differentiation of bronchoalveolar lavage fluid (BALF) from cats with FLAD.

	Eosinophilic inflammation (EI) (n = 6)	Neutrophilic inflammation (NI) (n = 2)	Mixed inflammation (MI) (n = 17)
**BALF total cell count (cells/μl)**	2585 (1235–5248)	1205 (1198–1213)	2810 (2470–3180)
**BALF eosinophils (%)**	60 (29–63)	10 (8–11)	48 (22–55)
**BALF neutrophils (%)**	3 (1.5–3)	47 (33–60)	12 (5–25)
**BALF macrophages (%)**	52 (37–74)	44 (32–55)	24 (12–38)
**BALF lymphocytes (%)**	0 (0–0)	0 (0–0)	0 (0–0)

Data are presented as median with IQR.

### Clinical 12-point score

The presenting clinical signs of cats on day 0 were coughing (n = 20; 80%), abnormal breathing sounds (n = 20; 80%) and episodes of respiratory distress (n = 14; 56%). The majority of the cats (n = 20; 80%) had abnormalities on thoracic auscultation on physical examination. General condition as well as appetite were reduced in eight cats (32%), one of which was classified as unresponsive/anorexic. The results of the clinical 12-point score at the three examination time points are shown in Table 4.

**Table 4:** Clinical 12-point score: Total clinical score and the individual criteria of the clinical 12-point score in cats with FLAD.

Clinical 12-point score	Day 0	Day 14	Day 60	p	p day 0–14	p day 0–60	p day 14–60
**Coughing frequency**	4 (1.5–5)	2 (0–2)	1 (0–2)	**0.009**	**<0.001**	**<0.001**	0.089
**Frequency of respiratory distress**	1 (0–2)	0 (0–0)	0 (0–0)	**0.001**	**<0.001**	**<0.001**	0.91
**Thoracic auscultation**	1 (1–1)	1 (0–1)	0 (0–1)	**0.002**	0.019	**<0.001**	0.16
**General condition and appetite**	0 (0–1)	0 (0–0)	0 (0–0)	**0.045**	0.40	**0.014**	0.094
**Total clinical score**	5 (4.5–7)	2 (1–5)	2 (0–3)	**<0.001**	**<0.001**	**<0.001**	**<0.001**

Data are presented as median with IQR. Bold values over the entire study period indicate p<0.05. The last three columns show the corrected values between the individual time periods. Bold values between individual time periods indicate p<0.017.

Throughout the study period, the total clinical score improved significantly over all three examination time points (p<0.001). Coughing frequency (p = 0.009), frequency of respiratory distress (p = 0.001), abnormal auscultation findings (p = 0.002) and the general condition and appetite of the cats (p = 0.045) responded significantly to therapy throughout the whole study period.

### Barometric whole-body plethysmography

All cats tolerated measurements well. The BWBP data of all cats generated at all three time points are listed in Table 5. Only the BWBP parameter *Penh* showed a significant change during the study period (p = 0.02). A significant difference was detected for Penh from day 0 to day 14 (p = 0.009), but not from day 14 to day 60 (p = 0.029); nor was the difference from day 0 to day 60 significant (p = 0.65). No significant changes could be detected for any of the other BWBP parameters over the study period.

**Table 5:** BWBP parameters at all three examination time points in cats with FLAD.

BWBP parameter	Unit	Day 0	Day 14	Day 60	p	p day 0–14	p day 0–60	p day 14–60
**RR**	breaths/min	61.8 (43.0–91.9)	59.6 (36.6–83.8)	61.2 (35.6–95.7)	0.59	n.a.		
**Ti**	s	0.4 (0.3–0.6)	0.4 (0.3–0.7)	0.4 (0.3–0.7)	0.59	n.a.		
**Te**	s	0.6 (0.4–0.9)	0.6 (0.5–1.1)	0.6 (0.4–1.0)	0.47	n.a.		
**TV**	ml	23.0 (14.2–33.5)	21.5 (15.6–27.6)	20.9 (16.2–29.4)	0.96	n.a.		
**TV/BW**	ml/kg	4.8 (3.0–7.3)	4.7 (3.2–8.0)	4.8 (3.4–6.8)	0.59	n.a.		
**MV**	ml/min	1138.5 (989.9–1687.1)	1192.4 (885.8–1487.9)	1238.8 (956.3–1658.5)	0.29	n.a.		
**MV/BW**	ml/min/kg	261.9 (242.2–345.2)	246.0 (201.2–301.2)	253.6 (212.6–311.1)	0.43	n.a.		
**PIF**	ml/s	75.8 (66.3–108.2)	78.5 (57.0–92.6)	73.2 (67.5–93.2)	0.29	n.a.		
**PIF/BW**	ml/s/kg	17.9 (13.9–22.7)	16.2 (13.5–20.8)	16.4 (13.7–19.4)	0.76	n.a.		
**PEF**	ml/s	49.0 (45.2–74.6)	48.6 (36.3–53.5)	46.4 (39.0–58.6)	0.29	n.a.		
**PEF/BW**	ml/s/kg	11.4 (9.2–16.2)	9.9 (9.0–11.2)	10.2 (8.0–14.3)	0.59	n.a.		
**EF50**	ml/s	41.6 (30.4–63.6)	38.7 (27.7–46.5)	40.1 (27.8–54.6)	0.70	n.a.		
**RT**	ms	0.3 (0.2–0.5)	0.4 (0.3–0.5)	0.3 (0.2–0.5)	0.14	n.a.		
**EIP**	ms	12.0 (10.4–14.9)	13.4 (11.7–18.1)	12.6 (10.7–15.8)	0.10	n.a.		
**EEP**	ms	3.9 (1.8–23.0)	6.3 (1.3–15.5)	3.0 (1.5–12.9)	0.62	n.a.		
**PAU**	unitless	0.9 (0.7–1.0)	0.8 (0.7–0.9)	0.8 (0.8–0.9)	0.59	n.a.		
**Penh**	unitless	0.7 (0.5–0.8)	0.5 (0.4–0.6)	0.5 (0.4–0.8)	**0.02**	**0.009**	0.65	0.03
**PEF/EF50**	unitless	1.2 (1.1–1.4)	1.2 (1.1–1.3)	1.2 (1.1–1.3)	0.47	n.a.		

Data are presented as median with IQR. Bold values indicate p<0.05. The last three columns show the corrected values between the individual time periods. Bold values between individual time periods indicate p<0.017. Not applicable (n.a.). Respiratory rate (RR; breaths/minute), inspiratory time (Ti; s), expiratory time (Te; s), tidal volume (TV; mL), tidal volume per body weight (TV/BW [mL/kg]), minute volume (MV; mL/min), minute volume per body weight (MV/BW; [mL/min/kg]), peak inspiratory pseudo-flow (PIF; mL/s), peak inspiratory pseudo-flow per body weight (PIF/BW; [mL/s/kg]), peak expiratory pseudo-flow (PEF; mL/s), peak expiratory pseudo-flow per body weight (PEF/BW; [mL/s/kg]), expiratory flow at end tidal volume plus 50% tidal volume (EF50; mL/s), relaxation time (RT; ms; time point when 65% of tidal volume is expired), end inspiratory pause (EIP; ms), end expiratory pause (EEP; ms), pause (PAU; unitless; (Te-RT)/RT), enhanced pause (Penh; unitless; (PEF/PIF)x(Te-RT)/RT) and quotient of peak expiratory pseudo-flow divided by expiratory flow at end expiratory volume plus 50% tidal volume (PEF/EF50; unitless).

### Correlation between the clinical 12-point score and BWBP parameters

The results of the investigation for correlation between the clinical 12-point score and the BWBP parameters for all three examination time points are illustrated in Tables 6–8.

**Table 6:** Correlation coefficients *r* between the clinical 12-point score and the individual BWBP parameters on day 0.

BWBP parameter day 0	Clinical 12-point score on day 0				
	Total clinical score	Coughing frequency	Frequency of respiratory distress	Thoracic auscultation	General condition / appetite
**RR**	**–0.35**	–0.11	–0.20	–0.20	–0.05
**Ti**	**0.30**	0.03	0.16	0.28	0.10
**Te**	0.29	0.11	0.09	0.19	–0.02
**TV**	0.20	0.01	0.09	0.22	0.08
**TV/BW**	0.18	0.02	0.08	0.20	0.03
**MV**	–0.10	–0.08	–0.04	0.05	0.02
**MV/BW**	–0.10	–0.16	–0.01	0.05	0.01
**PIF**	–0.07	–0.08	–0.01	0.01	0.10
**PIF/BW**	–0.01	–0.04	0.01	0.08	–0.03
**PEF**	–0.01	–0.14	0.12	0.27	0.17
**PEF/BW**	–0.04	–0.21	0.15	0.27	0.13
**EF50**	–0.06	–0.15	0.03	0.14	0.16
**RT**	0.28	0.13	0.06	0.10	–0.07
**EIP**	0.15	–0.05	0.13	0.19	0.30
**EEP**	0.21	0.04	0.05	0.27	0.24
**PAU**	0.22	0.05	0.03	**0.36**	0.08
**Penh**	0.15	–0.08	0.09	**0.40**	0.12
**PEF/EF50**	0.22	0.08	0.07	0.30	0.02

Bold values indicate p<0.05.

**Table 7:** Correlation coefficients *r* between the clinical 12-point score and the individual BWBP parameters on day 14.

BWBP parameter day 14	Clinical 12-point score on day 14				
	Total clinical score	Coughing frequency	Frequency of respiratory distress	Thoracic auscultation	General condition / appetite
**RR**	–0.19	–0.17	0.07	–0.12	–0.13
**Ti**	0.16	0.14	–0.06	0.14	0.07
**Te**	0.16	0.13	–0.07	0.05	0.08
**TV**	0.04	0.05	–0.03	0.16	0.01
**TV/BW**	0.01	0.00	–0.20	0.17	–0.01
**MV**	–0.23	-0.22	0.12	–0.08	–0.11
**MV/BW**	**–0.38**	**–0.40**	–0.15	–0.15	0.02
**PIF**	–0.25	–0.21	0.11	–0.13	–0.18
**PIF/BW**	**–0.42**	**–0.40**	–0.18	–0.24	–0.07
**PEF**	-0.26	–0.25	0.09	–0.06	–0.19
**PEF/BW**	**–0.48**	**–0.51**	–0.05	–0.10	–0.12
**EF50**	–0.20	0.19	0.16	–0.08	–0.14
**RT**	0.18	0.16	–0.06	0.09	0.09
**EIP**	0.13	0.06	0.08	0.16	0.08
**EEP**	–0.17	–0.15	–0.09	0.01	–0.15
**PAU**	0.01	0.10	–0.25	0.10	–0.18
**Penh**	–0.03	0.02	–0.11	0.16	–0.08
**PEF/EF50**	–0.04	–0.02	–0.12	0.08	–0.10

Bold values indicate p<0.05.

**Table 8:** Correlation coefficients *r* between the clinical 12-point score and the individual BWBP parameters on day 60.

BWBP parameter day 60	Clinical 12-point score on day 60				
	Total clinical score	Coughing frequency	Frequency of respiratory distress	Thoracic auscultation	General condition / appetite
**RR**	–0.06	–0.12	–0.26	0.10	–0.03
**Ti**	0.09	0.15	0.29	–0.08	0.07
**Te**	0.03	0.10	0.22	–0.13	0.02
**TV**	0.18	0.23	**0.41**	–0.06	0.20
**TV/BW**	0.05	0.10	**0.37**	–0.13	0.07
**MV**	0.23	0.16	0.13	0.25	0.26
**MV/BW**	0.06	–0.05	0.23	0.13	0.10
**PIF**	0.20	0.14	0.21	0.19	0.29
**PIF/BW**	–0.02	–0.14	0.29	–0.02	0.05
**PEF**	0.24	0.18	0.14	0.29	0.29
**PEF/BW**	–0.02	–0.09	0.18	0.06	0.05
**EF50**	0.22	0.15	0.06	0.27	0.24
**RT**	0.04	0.11	0.16	–0.12	–0.03
**EIP**	0.06	0.09	0.02	0.00	–0.12
**EEP**	–0.13	–0.07	0.10	–0.15	–0.03
**PAU**	–0.60	–0.01	**0.33**	–0.18	0.00
**Penh**	–0.02	–0.02	0.23	–0.02	0.02
**PEF/EF50**	–0.02	0.08	**0.34**	–0.23	0.07

Bold values indicate p<0.05.

## Discussion

The aim of this prospective study was to evaluate whether BWBP can be used as a non-invasive technique for therapeutic monitoring of cats with naturally occurring FLAD. The study reveals that the clinical 12-point score, as well as the BWBP parameter *Penh*, improved significantly under initial therapy.

Clinical examination is commonly used as the most important diagnostic tool to evaluate response to therapy in cats with FLAD [9]. In this study, the majority of cats showed coughing as a presenting complaint, which is consistent with previous reports [11, 17, 38, 51]. Abnormal respiratory sounds were described as frequently as coughing. Episodes of respiratory distress and pathological sounds on respiratory auscultation were also frequently noted, in agreement with published data [17, 38, 51]. In contrast to a previous study [38], the present investigation also detected a reduction in the general condition and appetite of 32% of the cats. This finding suggests that questions about the general condition and appetite of cats with respiratory issues should always be integrated into the anamnestic survey. The present study revealed a significant improvement in clinical signs and in thoracic auscultation findings under individualised anti-inflammatory therapy in cats with FLAD. A previous study showed a significant improvement in clinical signs in all of the 19 cats with naturally occurring FLAD that were available for reevaluation under therapy, after inhaling budesonide (200 μg; two puffs twice daily) over a period of two to 76 months [24]. Another study demonstrated a significant improvement in the total clinical score using a combined inhaled therapy of fluticasone (250 μg; two puffs twice daily) and salbutamol (100 μg; one to two puffs twice daily) over 25 to 219 days, while successively reducing the dosage of salbutamol [4]. In contrast to the present study, frequency of coughing and respiratory distress did not improve significantly in that study [4]. Nevertheless, the efficacy of inhaled glucocorticoid therapy using an inhalation chamber has been investigated by other authors and is considered an effective method for clinical improvement in cats with FLAD [26, 52]. In the present study, therapy was selected individually according to the severity of the disease and compliance of the cat and the owner, with the aim of a long-term conversion to inhalative therapy. Verschoor-Kirss and coworkers [53] compared the effect of oral and inhaled glucocorticoid therapy over an eight-week period in a randomised pilot study in nine cats with naturally occurring FLAD. While a group of four cats received 5 mg of prednisolone twice daily for 14 days, followed by 5 mg prednisolone once daily for the following six weeks, the other five cats were administered 5 mg of prednisolone once a day for seven days, followed by inhaled fluticasone (110 μg; twice daily) up to recheck. The authors reported complete elimination of clinical signs such as coughing or breathing difficulties in both groups [53]. A clinical score was not reported in that study, making comparability between study results challenging.

The BWBP parameter *Penh* improved significantly in the participating cats during the first 14 days. *Penh* is a calculated unitless parameter and is considered an indicator of bronchoconstriction in cats [43, 54]. The presence of increased airway resistance in cats with FLAD is an expected finding, likely due to the predominance of bronchoconstriction [2]. This may explain why appropriate therapy for FLAD resulted in significant improvement in *Penh* in this study. Previous studies used the parameter *Penh* primarily in the context of bronchoprovocation testing as *C-Penh-300*, as an indicator of airway hyperresponsiveness. *C-Penh-300* reflects the carbachol concentration that causes *Penh* to rise to 300% of the initial value [4, 26, 55–57]. The success of inhalative glucocorticoid therapy in cats with experimentally induced FLAD was evaluated by BWBP with bronchoprovocation testing in previous studies, in which *C-Penh-300* improved significantly under therapy [26, 58]. Bronchoprovocation testing to increase sensitivity of measurements during BWBP was not performed in this study due to concerns about potential risks and side effects. The use of bronchoprovocation as a routine method in client-owned cats is restricted due to the risk of severe bronchospasm [59]; nor should cats that already show bronchoconstriction during baseline measurement be subjected to bronchoprovocation testing [4, 43]. In one study, 16% of cats with FLAD showed signs of bronchospasm during basal *Penh* measurement, which made airway responsiveness testing in these cats non-justifiable [4]. Furthermore, it is known that carbachol, a commonly used bronchoprovocation agent, can increase the respiratory rate *RR*, which reduces the validity of BWBP parameters calculated from *RR*, including *Penh* [60]. In addition, the dosage of a bronchoprovocator agent strongly depends on the minute volume and body weight of the patient and can therefore be poorly compared between patients [61].

The significant improvement of *Penh* is consistent with results of a previous study in which *Penh* decreased significantly after therapy with inhalative budesonide (200 μg; two puffs twice daily) for at least two months in comparison to the pre-treatment status in 19 cats with naturally occurring FLAD [24]. In contrast to that, two other investigations failed to demonstrate a significant change in the parameter after therapy with corticosteroids +/- bronchodilators of client-owned cats with FLAD [4, 38]: one study showed no improvement in the parameter in 15 cats treated with oral prednisolone (1.2–2.0 mg/kg/day) or inhaled fluticasone (150–250 μg; q12), partly with addition of a bronchodilator (terbutaline or inhaled salbutamol), over a period of six months to three years [38]. However, in the second study, cats were treated with inhalative therapy only, consisting of a combination of fluticasone (250 μg; two puffs twice daily) and salbutamol (100 μg; one to two puffs twice daily) over 25 to 219 days [4]. This may explain the difference in the response of *Penh* in comparison to the present study, in which the majority of cats received systemic corticosteroids at least initially to control the inflammation in the airways as quickly as possible.

An increase of *Penh* after induced bronchoconstriction has been described for cats before [55, 57, 62–64]. Although *Penh* is widely accepted as an indicator of bronchoconstriction, it has been discussed controversially in the past by some authors [56, 65–69], as *Penh* is not a direct parameter of respiratory mechanics [70] and changes often do not correlate with airway resistance measured by pneumotachography and oesophageal balloon technique [56] or forced oscillation [65].

In this study, a significant decrease of *Penh* was detected between day 0 and day 14 only. This suggests that the initial therapy was the initiator of the improvement in airway resistance and thus in *Penh*. 88% of cats in this study received systemic anti-inflammatory agents for initial therapy, and later on treatment was adjusted to inhaled glucocorticoids as the sole long-term therapy to target airway inflammation locally with the lowest effective glucocorticoid dose. Initial systemic glucocorticoid therapy has positive effects on the inflammatory process [2], which induces airway and bronchial constriction, and this is reflected by the decrease of *Penh*. From day 14 to day 60, no further significant decrease of *Penh* could be detected. However, the mean value on day 60 was still lower than the pre-treatment value on day 0, while clinical signs continued to improve. As inhaled glucocorticoids were used for long-term therapy in most cats, BWBP on day 60 was undertaken under inhaled therapy in 96% of cats. A possible explanation for the finding that no further improvement of *Penh* was seen between day 14 and day 60 could be that the initially administered systemic glucocorticoids already led to a marked improvement of the pathological airway changes. After switching to inhaled glucocorticoids only, the difference between the prior effect on airway reactivity may have been too small to further influence *Penh*. As, to the authors’ knowledge, there are no published reference values for *Penh* in BWBP in healthy cats, and there was no healthy control group included in this study, it remains unclear how the values measured in this study under therapy relate to *Penh* in healthy cats. In a study, eight healthy cats were examined by BWBP and the mean values determined for *Penh* were in a range close to the values measured on day 14 in the present study [55]. Within cats treated with inhalative budesonide (200 μg; two puffs twice daily) for at least two months, patients showed values for *Penh* comparable to the mean values on day 60 in the present study [24]. Accordingly, it could also be possible that the measured values on day 14 and day 60 are already comparable to the values of healthy cats and thus further improvement is not measurable even by adequate therapy. However, since the cats’ clinical signs continued to improve after day 14, a positive therapeutic effect and ongoing control of the clinical signs was still visible.

As an additional point, it is well known that inhaled glucocorticoids can take one to two weeks to achieve maximal clinical improvement due to the delay in clinically effective absorption into the airway mucosa [2]. Thus, it is possible that systemic glucocorticoids may have already phased out before the inhaled glucocorticoids were fully flooded in. However, the continued clinical improvement illustrates that inhaled glucocorticoids also continue to control FLAD.

The reason why the clinical signs continued to improve, despite the lack of further improvement in *Penh* or other parameters, may also be due to the fact that the clinical signs shown in cats with FLAD are not induced by bronchial resistance only, but are also subject to many other pathological processes in the airways. If these improve, but the bronchial resistance is persistent, the clinical signs may continue to get better, without showing an improvement in *Penh*. It would have been interesting to monitor airway inflammation in addition to clinical signs and pulmonary function parameters, as the increased *Penh* on day 60 could suggest detection of clinically asymptomatic patients in that the prescribed treatment was not able to fully control the disease. Since performing repeated BALs for airway cytology would have implicated an unnecessary risk for the client-owned patients, a re-examination of airway inflammation was not carried out [71]. Subclinical inflammation and the detection of deterioration and lack of control of the disease pathology by BWBP on day 60 can therefore also not be ruled out.

In this study, *TV/BW* in cats with FLAD was below the normal range of 10–20 ml/kg [63]. It is not surprising for cats with FLAD to have a diminished *TV/BW* as hypoventilation is a consequence of chronic airway obstruction caused by the disease. The values measured for *TV/BW* did not change significantly in cats under therapy within the study period, which could suggest that obstruction is still at least partly present in the airways of the cats, even when they are under therapy. Low *TV/BW* values measured by BWBP in cats with FLAD, and without improvement under inhalative glucocorticoid therapy, have been reported in a previous study [4]. However, it is also known that *TV* is influenced by aspects other than ventilation, such as age, gender and obesity of the cat [54, 72]. A study investigating *TV* in BWBP in mice showed that *TV* can only be assessed qualitatively and not quantitatively by BWBP [73], restricting the interpretation of *TV/BW* and *TV* measurement in BWBP. In addition, Hoffman and coworkers [55] showed that reliable measurements of *TV* in BWBP are hampered by the effect of bronchoconstriction, and that BWBP only provides reliable values for *TV* in healthy cats. In a previous study, both cats with FLAD and healthy cats showed *TV/BW* values measured by BWBP below the reference range [39]. It is therefore not recommended that *TV* or *TV/BW* are used to grade the severity of the disease at diagnosis or monitor treatment response in cats with FLAD.

The comparison of results of BWBP measurements in cats with FLAD between studies is currently challenging due to the variation in study designs, such as the inclusion criteria of the study population, sample size, classification of types of airway inflammation and BWBP measurement protocols.

Another aim of the present study was to find out whether certain BWBP parameters correlate with the clinical score before and during therapy. Before starting individual therapy, the respiratory rate *RR* and the inspiratory time *Ti* measured in BWBP correlated moderately with the total clinical score. Cats with lower clinical scores showed higher values for *RR* and lower values for *Ti*. However, in a patient with mild clinical signs, a lower respiratory rate and a higher inspiratory time would be expected, compared to a more severely ill animal. The individual *RR* of a cat, however, does not depend exclusively on the presence of respiratory disease. As cats are known to be sensitive animals, an increase in respiratory rate can also be caused by other factors, such as fear or stress during veterinary examinations [74]. The higher the *RR*, the lower *Ti* will be as a consequence. In addition, the parameters *FF* and *Ti* are subject to circadian changes [54]. In a study comparing healthy cats and cats with FLAD, there were no significant differences between the BWBP values of *RR* and *Ti* between the two groups [39]. Therefore, the BWBP parameters *RR* and *Ti* should not be used to classify the severity of FLAD before therapy.

The severity of the detected auscultation findings correlated significantly with the BWBP parameters *Penh* and *PAU* prior to therapy. Since both of these parameters are considered indicators of bronchoconstriction, which in turn leads to abnormal auscultation sounds due to obstruction of the airways, it can be assumed that *Penh* and *PAU* could indeed serve as possible indicators of the severity of bronchoconstriction measured non-invasively. This information could help in determining whether a cat with FLAD should have a bronchodilator added to its individual treatment protocol.

Under initial therapy on day 14, the total clinical score and the coughing frequency correlated negatively with the BWBP parameters *MV/BW*, *PIF/BW* and *PEF/BW*, suggesting that more severe clinical signs (especially higher cough frequency) result in lower *MV/BW*, *PIF/BW* and *PEF/BW*. Lower minute volume, lower *PIF* and lower *PEF* in cats with FLAD can be explained by the obstructive character of the disease. In addition, previous studies were able to show that obesity can lead to a reduction in *MV*, *PIF* and *PEF* in cats as well [72]. Furthermore, measurements of *PIF* and *PEF* are also influenced by circadian changes [54].

On day 60, the frequency of respiratory distress showed a positive correlation with the BWBP parameters *PAU* and *PEF/EF50*. Similarly, Lin and coworkers [38] described a relationship between clinical signs and *PEF/EF50* in cats with FLAD. In that study, cats with higher *PEF/EF50* before and after glucocorticoid therapy had a longer history of disease and a higher respiratory distress score compared with cats that had lower values. As *PEF/EF50* and *PAU* are known to be obstruction-related parameters, the correlation with respiratory distress may be explained by greater expiratory airway obstruction.

Although most BWBP parameters, with the exception of *Penh*, did not improve significantly while the cats were undergoing therapy for FLAD in this study, it was possible to show that there is a relationship between certain BWBP parameters and the clinical score over the study period. A previous study showed an improvement of clinical signs in cats with FLAD during inhalative glucocorticoid therapy over 25 to 219 days with no correlation to an improvement of *Penh* or any other BWBP parameters [4]. A different study also failed to show that the clinical score is related to the presence of a concave exhalation curve [39]. Whether the severity of clinical signs can be reflected by BWBP parameters or whether the measurement of pulmonary function in BWBP may lag behind the clinical picture of cats with FLAD undergoing therapy requires larger investigations.

The limitations of this study mainly relate to the therapeutic schedule, as this was not standardised for all cats, but selected individually according to the severity of the disease and compliance of the cat, as well as the ability of the owner to administer medication. In addition, results may have been influenced by the subjectivity of the owners regarding the questionnaire that formed part of the clinical 12-point score. The authors had to rely on the owners’ objectivity and attentiveness in completing the survey.

As clinical signs in cats with FLAD often vary, it is difficult to determine improvement under therapy by clinical signs only [1, 2]. Repeated BAL while undergoing therapy would allow reliable evaluation of inflammation. However, performing repeated BAL was not considered justifiable in client-owned cats because of the risks associated with anaesthesia and the procedure.

## Conclusion

The results of this study support use of the BWBP parameter *Penh* as a non-invasive value for monitoring initial response to therapy in cats with FLAD. Clinical signs improved significantly under therapy over the entire study period, while temporarily correlating with certain BWBP parameters. Whether BWBP is suitable as a non-invasive tool for long-term monitoring in cats with naturally occurring FLAD, requires studies evaluating a longer period of time in the future.

## Supporting information

S1 Table Therapeutic agents administered for each individual cat between study time points. (DOCX)
