# Supplementary material for: Evaluation of barometric whole-body plethysmography for therapy monitoring in cats with feline lower airway disease
Source: PLoS One. 2022 Oct 27;17(10):e0276927. doi: 10.1371/journal.pone.0276927 (PMC9612479; doi:10.1371/journal.pone.0276927)
Supplement: S1 Table — (DOCX) [file pone.0276927.s001.docx]

**S1 Table. Therapeutic agents administered for each individual cat between study time points.**

| Cat number | Day 0 – Day 14 | Day 14 – Day 60 |
| --- | --- | --- |
| 1 | No therapy | Fluticasone propionate 250 µg |
| 2 | Terbutaline + Prednisolone | Partly Prednisolone in tapering +  Fluticasone propionate 250 µg |
| 3 | One-time Dexamethasone + Terbutaline + Prednisolone | Fluticasone propionate 250 µg |
| 4 | Terbutaline + Prednisolone +  Fluticasone propionate 250 µg | Fluticasone propionate 250 µg |
| 5 | Prednisolone + Budesonide 200 µg | Partly Prednisolone in tapering + Budesonide 200 µg |
| 6 | Prednisolone + Fluticasone propionate 250 µg | Fluticasone propionate 250 µg |
| 7 | Prednisolone | Fluticasone propionate 250 µg |
| 8 | Prednisolone | Fluticasone propionate 250 µg |
| 9 | Prednisolone + Fluticasone propionate 250 µg | Fluticasone propionate 250 µg |
| 10 | Prednisolone | Fluticasone propionate 250 µg |
| 11 | Prednisolone | Partly Prednisolone in tapering +  Fluticasone propionate 250 µg |
| 12 | Terbutaline + Prednisolone | Fluticasone propionate 250 µg |
| 13 | Terbutaline + Prednisolone +  Fluticasone propionate 250 µg | Fluticasone propionate 250 µg |
| 14 | Terbutaline + Prednisolone | Fluticasone propionate 250 µg |
| 15 | Terbutaline + Fluticasone propionate 250 µg | Fluticasone propionate 250 µg |
| 16 | Prednisolone | Partly Prednisolone in tapering +  Fluticasone propionate 250 µg |
| 17 | Terbutaline + Prednisolone | Fluticasone propionate 250 µg |
| 18 | Terbutaline + Prednisolone +  Fluticasone propionate 250 µg | Fluticasone propionate 250 µg |
| 19 | Prednisolone | Fluticasone propionate 250 µg |
| 20 | Prednisolone | Fluticasone propionate 250 µg |
| 21 | Terbutaline | Salmeterol and fluticasone propionate 25 µg/125 µg |
| 22 | One-time Dexamethason + Prednisolone | Partly Prednisolone in tapering +  Fluticasone propionate 250 µg |
| 23 | Cyclosporine + Fluticasone propionate 125 µg | Cyclosporine + Fluticasone propionate 125 µg |
| 24 | One-time Dexamethasone + Prednisolone | Fluticasone propionate 250 µg |
| 25 | No therapy | Prednisolone |
